# Immunogenicity and Safety of Coadministration of AS01_E_-Adjuvanted Respiratory Syncytial Virus Prefusion F Protein Vaccine and a COVID-19 mRNA Vaccine in Adult s Aged ≥50 Years: A Phase 3, Randomized, Noninferiority Trial

**DOI:** 10.1093/cid/ciag344

**Published:** 2026-06-04

**Authors:** Marc Bonten, Brandon Essink, Nikita Hanning, Isabel Leroux-Roels, Cristina Masuet-Aumatell, Silvina Natalini Martinez, Hiwot Amare Hailemariam, Ghassem Jandari, Paulia Kwo Ton Ge Jua, Charlotte Servais, Sofía Valenciano, Archana M Jastorff, Vicky Cárdenas, Marie Van der Wielen

**Affiliations:** Julius Center for Health Sciences and Primary Care, University Medical Centre Utrecht, Utrecht University, Utrecht, The Netherlands; European Clinical Research Alliance on Infectious Diseases (ECRAID), Utrecht, The Netherlands; Velocity Clinical Research, Omaha, Nebraska, USA; Centre for the Evaluation of Vaccination, Vaccine and Infectious Disease Institute (VAXINFECTIO), University of Antwerp, Antwerp, Belgium; Center for Vaccinology, Ghent University and Ghent University Hospital, Ghent, Belgium; Preventive Medicine Department, University Hospital Bellvitge, IDIBELL, L’Hospitalet de Llobregat, Barcelona, Spain; Hospital Universitario HM Puerta del Sur, Unidad de Investigación de Vacunas, Instituto de Investigación Sanitaria HM, Madrid, Spain; GSK, Wavre, Belgium; GSK, Wavre, Belgium; GSK, Wavre, Belgium; GSK, Rixensart, Belgium; GSK, Wavre, Belgium; GSK, Wavre, Belgium; GSK, Rockville, Maryland, USA; GSK, Wavre, Belgium

**Keywords:** adjuvanted RSVPreF3, COVID-19 mRNA vaccine, coadministration, immunogenicity, safety

## Abstract

**Background:**

This trial evaluated coadministration of the AS01_E_-adjuvanted respiratory syncytial virus (RSV) prefusion F protein-based vaccine (adjuvanted RSVPreF3) and an Omicron XBB.1.5-based coronavirus disease 2019 (COVID-19) mRNA vaccine in ≥50-year-olds.

**Methods:**

This phase 3, open-label, multicenter trial randomized ≥50-year-olds 1:1 to coadministration (Co-Ad group) or sequential administration approximately 1 month apart (Control group) of adjuvanted RSVPreF3 and the COVID-19 mRNA vaccine. Primary objectives were to demonstrate noninferiority of humoral immune responses at 1 month postvaccination in terms of RSV-A, RSV-B, and severe acute respiratory syndrome coronavirus 2 (SARS-CoV-2) neutralizing titers, with the upper limit of the 95% CI of adjusted geometric mean titer (GMT) group ratios (Control/Co-Ad) ≤1.50. Secondary objectives included assessment of reactogenicity up to 4 days and safety up to 6 months after vaccination.

**Results:**

Overall, 833 participants were vaccinated (Co-Ad: 417; Control: 416). Noninferiority was achieved for RSV-A (GMT ratio: 1.12 [95% CI: .97–1.28]) and RSV-B (GMT ratio: 1.08 [95% CI: .94–1.23]) and marginally missed for SARS-CoV-2 Omicron XBB.1.5 (GMT ratio: 1.31 [95% CI: 1.13–1.51]). Within 4 days postvaccination, the most frequently reported solicited events were administration-site pain, myalgia, and fatigue. Most solicited events were of mild-to-moderate intensity, and the overall median duration was ≤3 days and comparable between groups. Unsolicited and serious adverse events were generally balanced between groups.

**Conclusions:**

Adjuvanted RSVPreF3 coadministered with a COVID-19 mRNA vaccine maintained an acceptable safety profile in ≥50-year-olds. The marginal miss of the noninferiority criterion for SARS-CoV-2 does not suggest clinically relevant interference. Therefore, the results support the coadministration of these vaccines.

**Clinical Trials Registration:**

Clinical Trials.gov. NCT06374394.

## Teaser

Coadministering the AS01_E_-adjuvanted RSVPreF3 vaccine and a COVID-19 mRNA vaccine in ≥50-year-olds elicited neutralizing antibody responses without clinically relevant immune interference to either vaccine and maintained an acceptable safety profile up to 6 months after vaccination.

Respiratory syncytial virus (RSV) is a major cause of respiratory infections worldwide [1], with seasonal epidemics in temperate climates occurring in late fall and winter [1, 2]. Although infants are at greatest risk of severe RSV-related outcomes [3], adults also experience a substantial burden, with hospitalization and mortality rates increasing with age [4, 5]. A meta-analysis estimated approximately 42 000 RSV-attributed hospitalizations annually in adults aged 50–64 years and 159 000 in those aged ≥65 years in the United States [4]. Adults who are immunocompromised and those with chronic medical conditions (eg, chronic cardiopulmonary diseases, diabetes, and chronic renal and liver diseases) are also at increased risk of severe RSV disease [5–7].

Coronavirus disease 2019 (COVID-19) also contributes to the burden of respiratory infections in adults [8], especially among older adults and those with certain medical conditions, including immunocompromised individuals [9]—populations that overlap with those at risk of RSV. Although severe acute respiratory syndrome coronavirus 2 (SARS-CoV-2) circulates year-round, seasonal spikes in COVID-19-related hospitalizations and deaths, most prominent during the winter season [10], often align with RSV seasonality. Vaccination remains a cornerstone of protection against severe COVID-19 for high-risk groups, with updated, variant-adapted vaccine versions being offered through routine or targeted immunization strategies [11, 12], often alongside those for seasonal influenza and RSV.

Given the overlap in at-risk populations and seasonality for RSV and SARS-CoV-2, coadministration of RSV and COVID-19 vaccines could improve adherence to vaccination recommendations, increase vaccine uptake, and reduce healthcare visits and associated costs [13].

The AS01_E_-adjuvanted RSV prefusion F protein-based vaccine (adjuvanted RSVPreF3; *Arexvy*, GSK) has demonstrated efficacy against RSV-related lower respiratory tract disease over 3 RSV seasons and has shown an acceptable safety profile in adults ≥60 years [14]. The vaccine also showed noninferior immunogenicity and a consistent safety profile in 18–59-year-olds [15–17]. It was initially approved for adults ≥60 years and for adults 50–59 years at increased risk of RSV disease in the United States, Europe, and other regions, with a recent expansion of its indication to adults ≥18 years at increased risk of RSV disease in the United States and to all adults ≥18 years in Europe [18, 19]. Real-world evidence confirms trial findings, with observed reductions in RSV-related hospitalizations in adults ≥60 years [20–22]. Prior studies have reported acceptable reactogenicity and safety profiles of coadministration of adjuvanted RSVPreF3 with influenza [23–25] and zoster [26] vaccines, without clinically relevant interference of immunogenicity. There are currently no data on the coadministration of adjuvanted RSVPreF3 with COVID-19 mRNA vaccines, leaving an evidence gap for clinical decision-making and for informing vaccine policy.

In this study, we evaluated the immunogenicity, safety, and reactogenicity of coadministration of adjuvanted RSVPreF3 with an Omicron XBB.1.5-based COVID-19 mRNA vaccine (*Comirnaty*, BioNTech). The SARS-CoV-2 XBB.1.5 variant predominated in 2023 [27, 28] and XBB.1.5-based vaccines were widely recommended for the 2023–2024 COVID-19 season [29], when enrollment in this study started.

The primary study objective was to demonstrate noninferiority of humoral immune responses 1 month after coadministration of the vaccines compared with their sequential administration approximately 1 month apart in adults ≥50 years. Secondary endpoints included other immunogenicity measures of both vaccines, reactogenicity, and safety.

Figure 1 presents a study summary in plain language.

**Figure 1.**
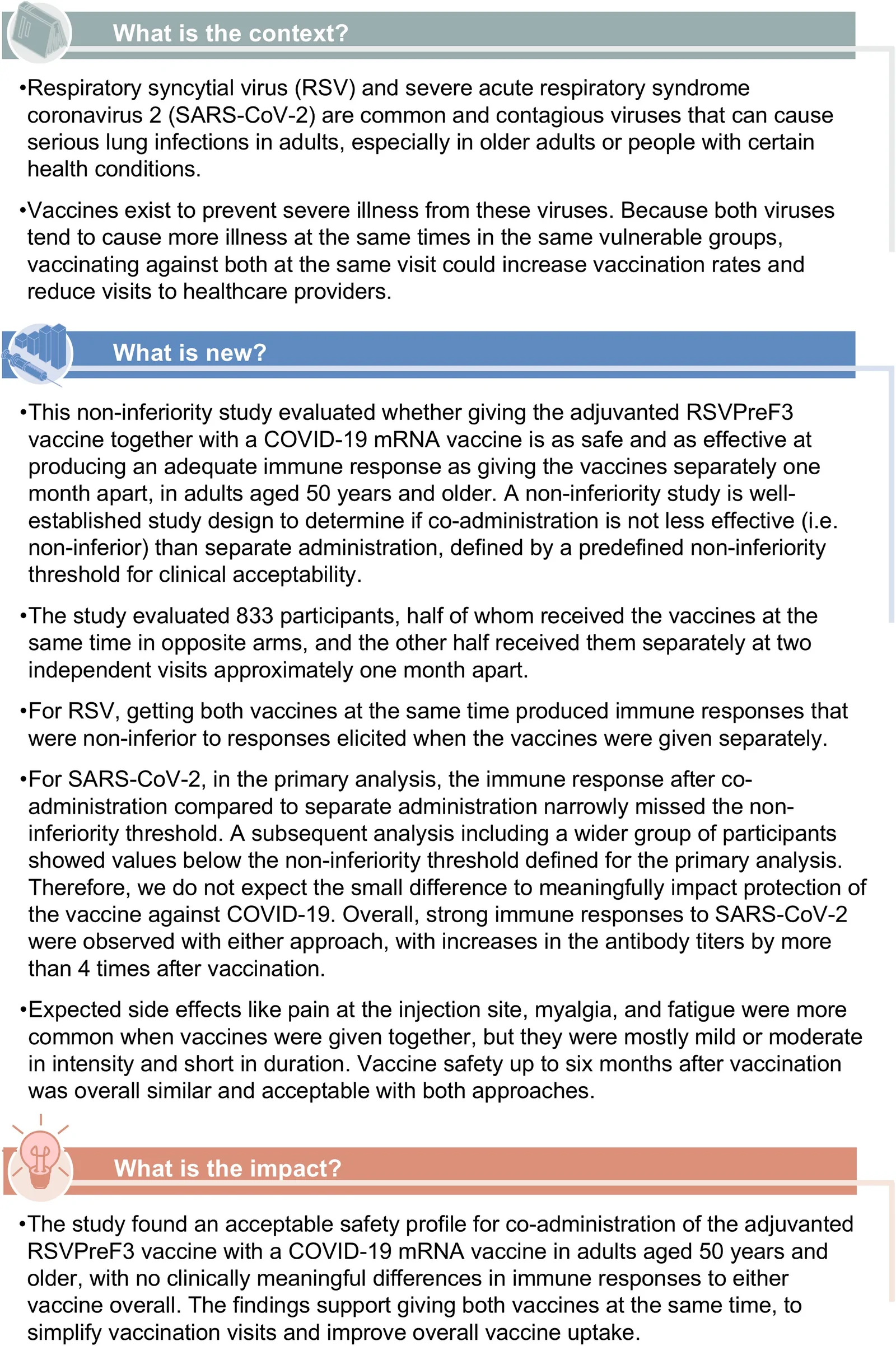
Plain language summary.

## METHODS

### Study Design, Participants, and Interventions

This phase 3, open-label, randomized, controlled trial (NCT06374394) was conducted between 29 April 2024 and 18 April 2025 at 24 centers in the United States, Belgium, the Netherlands, and Spain.

The study included adults ≥50 years, including those with stable chronic conditions. Individuals with confirmed or suspected immunosuppressive or immunodeficient conditions resulting from disease or treatment were not eligible to participate. Participants were required to have received at least one COVID-19 vaccine 3 or more months prior to study start and could not have had a SARS-CoV-2 infection within the 3 months prior to COVID-19 vaccine administration in this study. Participants who previously received an RSV vaccine were ineligible to participate.

Participants were randomized 1:1 to either the Coadministration (Co-Ad) or Control group (Figure 2). In the Co-Ad group, participants received a single dose of the COVID-19 mRNA vaccine and a single dose of adjuvanted RSVPreF3 at Day 1. In the Control group, participants received the COVID-19 mRNA vaccine at Day 1 and adjuvanted RSVPreF3 approximately 1 month later (Day 31).

**Figure 2.**
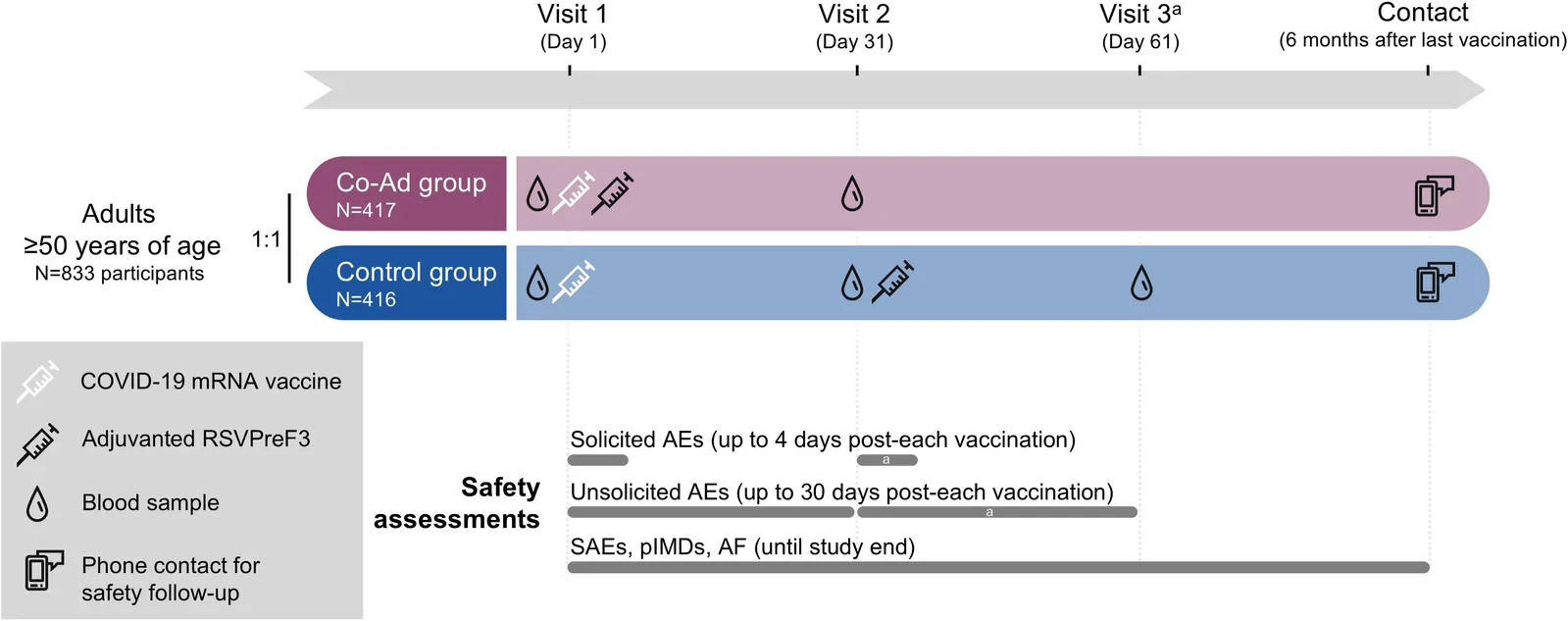
Study design. Co-Ad, group of participants receiving a single dose of a coronavirus disease 2019 (COVID-19) mRNA vaccine and a single dose of AS01_E_-adjuvanted respiratory syncytial virus (RSV) prefusion F protein-based vaccine (adjuvanted RSVPreF3) at Day 1; Control, group of participants receiving a single dose of a COVID-19 mRNA vaccine at Day 1, followed by a single dose of adjuvanted RSVPreF3 at Day 31; N, number of participants in the exposed population; AE, adverse event; SAE, serious AE; pIMD, potential immune-mediated disease; AF, atrial fibrillation. ^a^Applies to the Control group only.

The standard dose of both vaccines was administered intramuscularly in the deltoid region of either the nondominant (COVID-19 mRNA vaccine in both groups; adjuvanted RSVPreF3 in the Control group) or dominant arm (adjuvanted RSVPreF3 in the Co-Ad group).

This was an open-label study due to the obvious difference in dosing schedules between groups. Potential bias was reduced through central randomization and blinding of the laboratory staff conducting blood sample immunogenicity testing.

Eligibility criteria, enrollment rules, and randomization procedures are detailed in the Supplementary Methods.

The study was conducted in accordance with the Declaration of Helsinki, Good Clinical Practice guidelines, and applicable regulatory requirements, and adhered to the study protocol as approved by relevant ethics committees (summary: https://www.gsk-studyregister.com/en/trial-details/?id=217848). All participants provided written informed consent.

### Objectives

Primary objectives were to demonstrate noninferiority of the immune responses to adjuvanted RSVPreF3 and the COVID-19 mRNA vaccine when coadministered compared with when administered approximately 1 month apart, in terms of RSV-A, RSV-B, and SARS-CoV-2 Omicron XBB.1.5 geometric mean titer (GMT) ratios 1 month after each vaccination.

Secondary endpoints included evaluation of additional descriptive immunogenicity measures (ie, mean geometric increase [MGI] and seroresponse rate [SRR]) of both vaccines at 1 month, reactogenicity within 4 days, and safety within 6 months postvaccination.

### Immunogenicity Assessments

Blood samples were collected from all participants at baseline (Day 1) and at 1 month after (first) vaccination (Day 31). From participants in the Control group, an additional sample was collected at 1 month after the last vaccination (Day 61) (Figure 2).

Respiratory syncytial virus-A and RSV-B neutralizing titers were measured using an in-house neutralization assay and expressed in estimated dilution 60 (ED60) [30]. Serum SARS-CoV-2 Omicron XBB.1.5 neutralization titers were assessed using a pseudotyped virus neutralization assay and expressed in the dilution corresponding to 50% signal reduction (NT50) (Supplementary Methods). Assay cutoff values were 18 ED60 (RSV-A), 30 ED60 (RSV-B), and 15 NT50 (SARS-CoV-2).

### Reactogenicity and Safety Assessments

Participants recorded solicited events that started within 4 days and unsolicited adverse events (AEs) that started within 30 days after vaccination using paper diary cards or through an interview at the next visit (for unsolicited AEs). Serious AEs (SAEs) and AEs of special interest (AESIs), including potential immune-mediated diseases (pIMDs) and atrial fibrillation (AF), were collected up to study end (6 months postvaccination) through inquiry at study visits/contacts. The intensity of all solicited events, unsolicited AEs, AESIs, and SAEs was graded as mild (grade 1), moderate (grade 2), or severe (grade 3). Solicited events were graded by participants, and unsolicited AEs, AESIs, and SAEs by the investigator. The investigator assessed the relationship of unsolicited AEs, AESIs, and SAEs with study vaccinations and determined the outcome of all AEs.

### Statistical Methods

The aim was to enroll approximately 830 participants (Supplementary Methods). Immunogenicity objectives were analyzed on the applicable per-protocol sets (ie, RSV per-protocol set and COVID-19 mRNA per-protocol set). These sets included all eligible participants who received at least one (Control) or both (Co-Ad) study vaccinations as per protocol, had both pre- and postvaccination immunogenicity results, complied with blood draw intervals, had no intercurrent medical conditions that may interfere with immunogenicity, did not receive a prohibited concomitant medication or vaccination, and did not meet any of the elimination criteria up to the blood sampling timepoints. Because the percentage of vaccinated participants with immunogenicity results excluded from each per-protocol set was >5% for the noninferiority analysis, a sensitivity analysis based on the exposed population (ie, all participants who received a study vaccine) was performed to complement the per-protocol set analyses, as prespecified in the protocol.

The study included 3 primary confirmatory noninferiority endpoints. Noninferiority was demonstrated when the upper limit of the 2-sided 95% CI of the GMT ratios between the Control versus the Co-Ad group for RSV-A, RSV-B, and SARS-CoV-2 neutralizing titers at 1 month after each vaccination was ≤1.50. The CIs were derived from an analysis of covariance model on log_10_-transformed titers for each neutralization assay, with the study group and age category (50–59, 60–69, or ≥70 years) as fixed effects and the baseline log_10_-transformed titer as a covariate. Missing data were not replaced. Titers below the assay lower limit of quantification (LLOQ) were replaced by half the LLOQ; titers above the upper limit of quantification (ULOQ) were replaced by the ULOQ.

Respiratory syncytial virus-A, RSV-B, and SARS-CoV-2 neutralizing GMTs, MGIs (ie, geometric mean of the within-participant ratios 1 month after each vaccination over baseline), and SRRs (ie, proportion of participants with a ≥4-fold increase in neutralizing titers compared with baseline) were calculated with 95% CIs. Descriptive immunogenicity analyses were also performed by age category (50–59, 60–69, and ≥70 years).

Demographic characteristics and safety outcomes were analyzed on the exposed population and summarized using descriptive statistics. Reactogenicity outcomes were also summarized by age category.

Statistical analyses were performed using SAS v9.4 or higher (SAS Institute Inc., Cary, NC, USA).

## RESULTS

### Study Participants

In total, 833 participants (Co-Ad: 417; Control: 416) received adjuvanted RSVPreF3 and the COVID-19 mRNA vaccine, and 807 (96.9%) participants completed the study (Figure 3). The RSV per-protocol set for primary analysis included 382 (91.6%) Co-Ad and 364 (87.5%) Control participants and the COVID-19 mRNA per-protocol set for primary analysis included 380 (91.1%) Co-Ad and 381 (91.6%) Control participants. The main reason for elimination from the per-protocol sets was out-of-window timepoint assessment for immunogenicity (4.6% across groups for RSV per-protocol set and 2.2% for COVID-19 mRNA per-protocol set).

**Figure 3.**
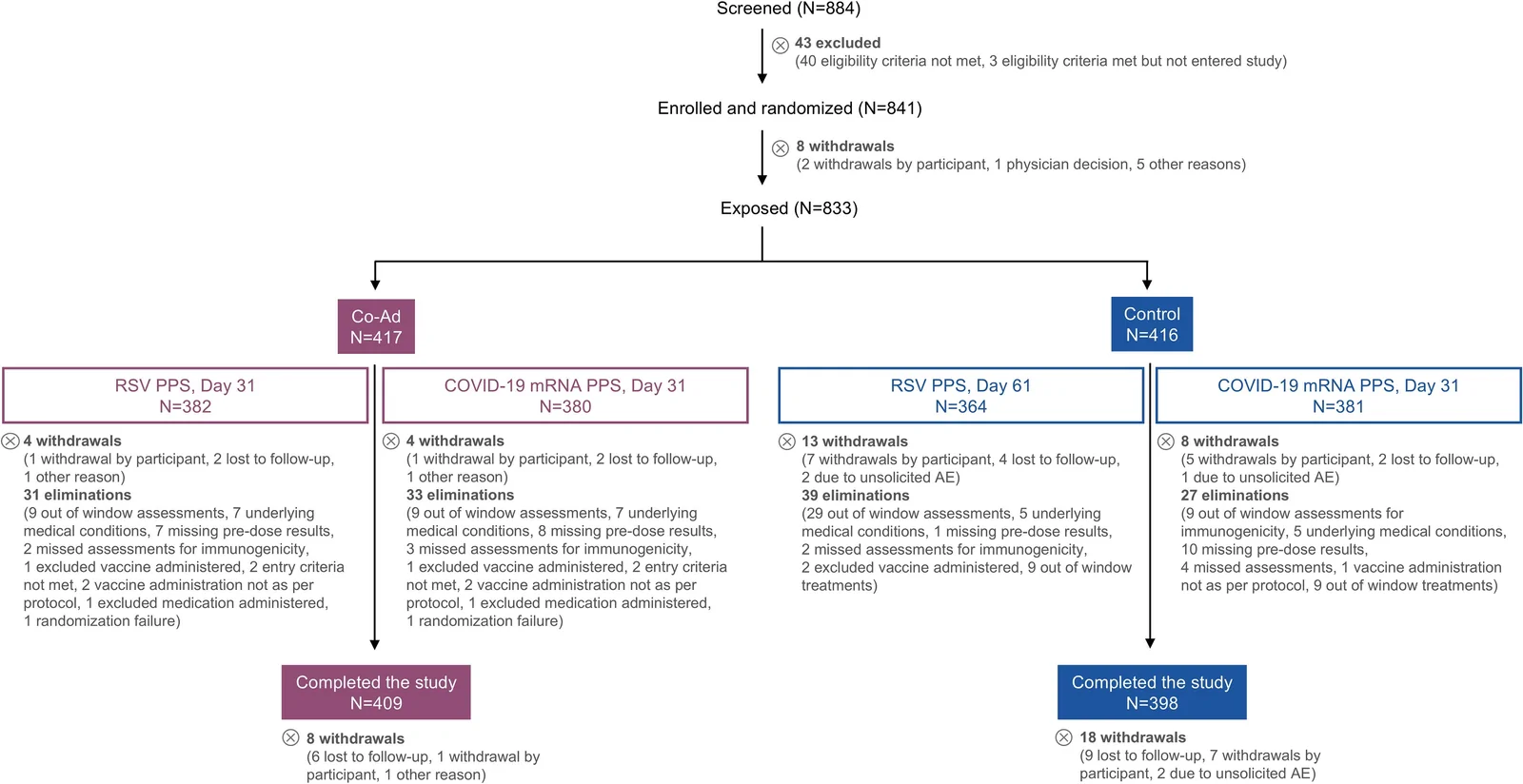
Participant flow. Co-Ad, group of participants receiving a single dose of a coronavirus disease 2019 (COVID-19) mRNA vaccine and a single dose of AS01_E_-adjuvanted respiratory syncytial virus (RSV) prefusion F protein-based vaccine (adjuvanted RSVPreF3) at Day 1; Control, group of participants receiving a single dose of a COVID-19 mRNA vaccine at Day 1, followed by a single dose of adjuvanted RSVPreF3 at Day 31; PPS, per-protocol set; N, number of participants in the indicated analysis set and group; AE, adverse event. Note: For eliminations from the per-protocol sets, the total number of eliminated participants are shown. Multiple reasons for eliminations could apply for 1 participant; all reasons are listed between brackets.

Overall, baseline characteristics of the participants were balanced between groups in the exposed population (Table 1) and in both per-protocol sets (Supplementary Table 1). The proportion of participants reporting an underlying medical condition was similar between groups (Co-Ad: 91.8%; Control: 95.0%). The 3 most frequently reported conditions based on MedDRA System Organ Class classification were metabolism and nutrition disorders (49.4%; 47.8%), vascular disorders (46.0%; 42.3%), and musculoskeletal and connective tissue disorders (32.6%; 26.2%) (Supplementary Table 2).

**Table 1.** Baseline Characteristics of the Participants, Exposed Population

Characteristic	Co-Ad N = 417	Control N = 416
Mean (SD) age at vaccination, y	63.7 (8.4)	63.5 (8.4)
Age category, n (%)		
50–59 y	154 (36.9)	159 (38.2)
60–69 y	149 (35.7)	145 (34.9)
≥70 y	114 (27.3)	112 (26.9)
≥80 y	16 (3.8)	8 (1.9)
Female sex, n (%)	216 (51.8)	223 (53.6)
Race, n (%)		
Black or African American	31 (7.4)	32 (7.7)
White	378 (90.6)	375 (90.1)
Other^a^	8 (2.0)	9 (2.2)
Ethnicity, n (%)		
Hispanic or Latino	29 (7.0)	23 (5.5)
Not Hispanic or Latino	383 (91.8)	390 (93.8)
Not reported	5 (1.2)	3 (0.7)
Country, n (%)		
Belgium	119 (28.5)	122 (29.3)
The Netherlands	69 (16.5)	78 (18.8)
Spain	103 (24.7)	89 (21.4)
United States	126 (30.2)	127 (30.5)

Abbreviations: Co-Ad, group of participants receiving a single dose of a COVID-19 mRNA vaccine and a single dose of AS01_E_-adjuvanted respiratory syncytial virus (RSV) prefusion F protein-based vaccine (adjuvanted RSVPreF3) at Day 1; Control, group of participants receiving a single dose of a coronavirus disease 2019 (COVID-19) mRNA vaccine at Day 1, followed by a single dose of adjuvanted RSVPreF3 at Day 31; N, number of participants in the exposed population; SD, standard deviation; y, years; n (%), number (percentage) of participants in a given category.

^a^Includes American Indian or Alaska Native, Asian, multiple race categories, or race not reported.

### Immunological Noninferiority: Primary Endpoint

Approximately 1 month after coadministration of adjuvanted RSVPreF3 and the COVID-19 mRNA vaccine, the primary analysis on the RSV per-protocol set demonstrated noninferior immune responses in terms of RSV neutralizing titers compared with when adjuvanted RSVPreF3 was administered sequentially with the COVID-19 mRNA vaccine, with the upper limits of the 95% CIs for group GMT ratios ≤1.50 for both RSV-A (GMT ratio: 1.12 [95% CI: .97–1.28]) and RSV-B (GMT ratio: 1.08 [95% CI: .94–1.23]) (Figure 4*A*). For SARS-CoV-2 Omicron XBB.1.5, the primary confirmatory immunogenicity endpoint was marginally missed, with an upper limit of 1.51 (GMT ratio: 1.31 [95% CI: 1.13–1.51]) (Figure 4*A*).

**Figure 4.**
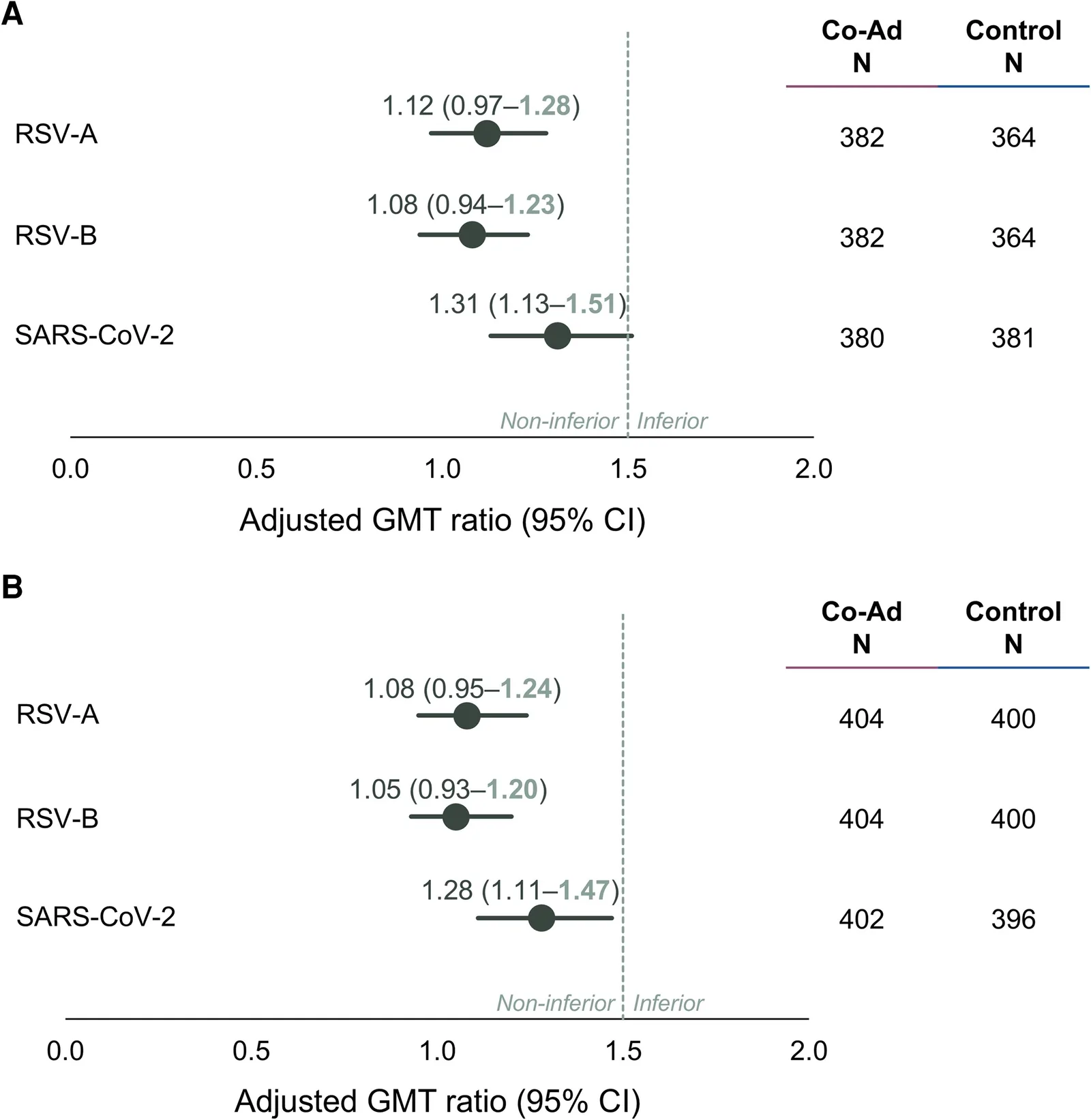
Noninferiority of the humoral immune response to adjuvanted RSVPreF3 and coronavirus disease 2019 (COVID-19) mRNA vaccine when coadministered compared with when administered sequentially at 1 month postvaccination, on (*A*) the RSV and COVID-19 mRNA per-protocol sets for primary analysis, and (*B*) the exposed population. RSV, respiratory syncytial virus; SARS-CoV-2, severe acute respiratory syndrome coronavirus 2; Co-Ad, group of participants receiving a single dose of a COVID-19 mRNA vaccine and a single dose of AS01_E_-adjuvanted RSV prefusion F protein-based vaccine (adjuvanted RSVPreF3) at Day 1; Control, group of participants receiving a single dose of a COVID-19 mRNA vaccine at Day 1, followed by a single dose of adjuvanted RSVPreF3 at Day 31; N, number of participants in the applicable per-protocol set; GMT, geometric mean titer. Notes: Noninferiority was demonstrated if the upper limit of the 2-sided 95% CIs was ≤1.50 for the group GMT ratios (Control over Co-Ad) for RSV-A, RSV-B, and SARS-CoV-2 at 1 month postvaccination in the per-protocol set. Two-sided 95% CIs are depicted as error bars, and noninferiority margins with a dashed line.

Because >5% of participants were excluded from the per-protocol sets in both groups, sensitivity analyses were conducted in the larger exposed population, as required per protocol. These analyses showed upper limits of the 95% CIs for group GMT ratios ≤1.50 for RSV-A, RSV-B, and SARS-CoV-2 Omicron XBB.1.5 (Figure 4*B*).

### Immunogenicity for Adjuvanted RSVPreF3

Descriptive analyses of immunogenicity for adjuvanted RSVPreF3 were performed as a secondary endpoint. At baseline, all participants had RSV-A and RSV-B neutralizing titers equal to or above the respective assay cutoffs, presumably due to previous RSV exposure. Baseline GMTs for both RSV subtypes tended to be lower in the Control group compared with the Co-Ad group.

One month postvaccination, RSV-A and RSV-B neutralizing titers strongly increased in both groups compared with baseline, with MGIs of 8.31 (95% CI: 7.46–9.26) for RSV-A and 6.79 (95% CI: 6.08–7.59) for RSV-B in the Co-Ad group and 10.21 (95% CI: 9.20–11.33) for RSV-A and 8.19 (95% CI: 7.35–9.12) for RSV-B in the Control group (Figure 5). In both groups, immunogenicity across age categories was consistent with that observed in the overall population (Supplementary Figure 1).

**Figure 5.**
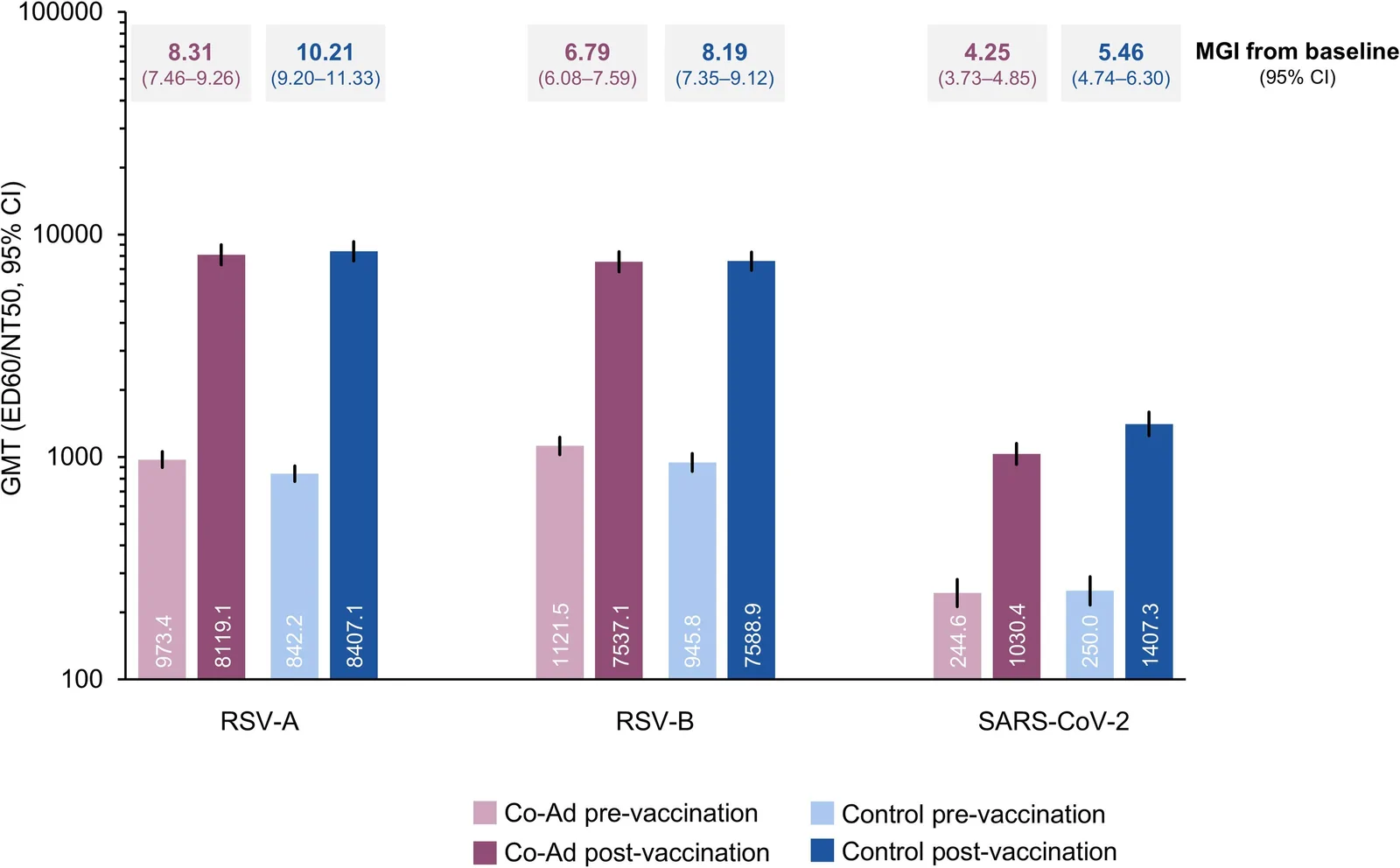
RSV-A, RSV-B, and SARS-CoV-2 neutralizing titers after adjuvanted RSVPreF3 and coronavirus disease 2019 (COVID-19) mRNA vaccine coadministration or sequential administration, RSV, and COVID-19 mRNA per-protocol sets. RSV, respiratory syncytial virus; SARS-CoV-2, severe acute respiratory syndrome coronavirus 2; Co-Ad, group of participants receiving a single dose of a COVID-19 mRNA vaccine and a single dose of AS01_E_-adjuvanted RSV prefusion F protein-based vaccine (adjuvanted RSVPreF3) at Day 1; Control, group of participants receiving a single dose of a COVID-19 mRNA vaccine at Day 1, followed by a single dose of adjuvanted RSVPreF3 at Day 31; GMT, geometric mean titer; ED60, estimated dilution 60; NT50, serum dilution corresponding to 50% signal reduction; MGI, mean geometric increase postvaccination compared with prevaccination. Note: For GMTs, the number of participants in the RSV per-protocol set with available results were 406 Co-Ad prevaccination, 388 Co-Ad postvaccination, 389 Control prevaccination, 365 Control postvaccination, and the number of participants in the COVID mRNA per-protocol set were 405 Co-Ad prevaccination, 387 Co-Ad postvaccination, 404 Control prevaccination, and 389 Control postvaccination. For MGIs, the number of participants in the RSV per-protocol set with available results at both timepoints were 382 Co-Ad and 364 Control and in the COVID-19 mRNA per-protocol set were 380 Co-Ad and 381 Control.

Seroresponse rates for RSV neutralizing titers 1 month after adjuvanted RSVPreF3 administration were comparable across groups. For RSV-A, SRRs were 73.8% (95% CI: 69.1–78.2) in the Co-Ad and 81.3% (76.9–85.2) in the Control group, and for RSV-B, SRRs were 66.8% (61.8–71.5) and 75.5% (70.8–79.9), respectively.

### Immunogenicity for COVID-19 mRNA Vaccine

Descriptive analyses of immunogenicity for the COVID-19 mRNA vaccine were performed as a secondary endpoint. At baseline, 95.1% of participants in the Co-Ad and 94.3% in the Control group had SARS-CoV-2 Omicron XBB.1.5 neutralizing titers equal to or above the assay cutoff, presumably due to previous SARS-CoV-2 exposure.

In both groups, enhanced SARS-CoV-2 neutralizing titers were observed 1 month postvaccination compared with baseline, with MGIs of 4.25 (95% CI: 3.73–4.85) in the Co-Ad and 5.46 (95% CI: 4.74–6.30) in the Control group (Figure 5). Geometric mean titers were higher in the Control group than in the Co-Ad group. Age-based subgroup analysis indicated that immunogenicity in each age category was consistent with that observed in the overall population in both groups (Supplementary Figure 1).

To further characterize the SARS-CoV-2 immune response, SRRs at 1 month postvaccination were calculated (post hoc testing), and these were 41.6% (36.6–46.7) in the Co-Ad group and 49.3% (44.2–54.5) in the Control group.

### Reactogenicity and Safety

Solicited events were reported by 87.4% of Co-Ad and 81.0% of Control participants after any vaccine administration.

In both groups, the most common solicited administration-site event was pain, both at the adjuvanted RSVPreF3 site (Co-Ad: 73.3%; Control: 58.5%) and COVID-19 mRNA vaccine site (Co-Ad: 72.3%; Control: 64.2%) (Figure 6). The most frequently reported solicited systemic events were myalgia (Co-Ad: 41.3%; Control post-COVID-19 mRNA vaccine: 28.5%; Control postadjuvanted RSVPreF3: 22.6%) and fatigue (40.3%, 28.0%, and 23.6%, respectively). Frequencies of solicited systemic events per participant are shown in Supplementary Table 3.

**Figure 6.**
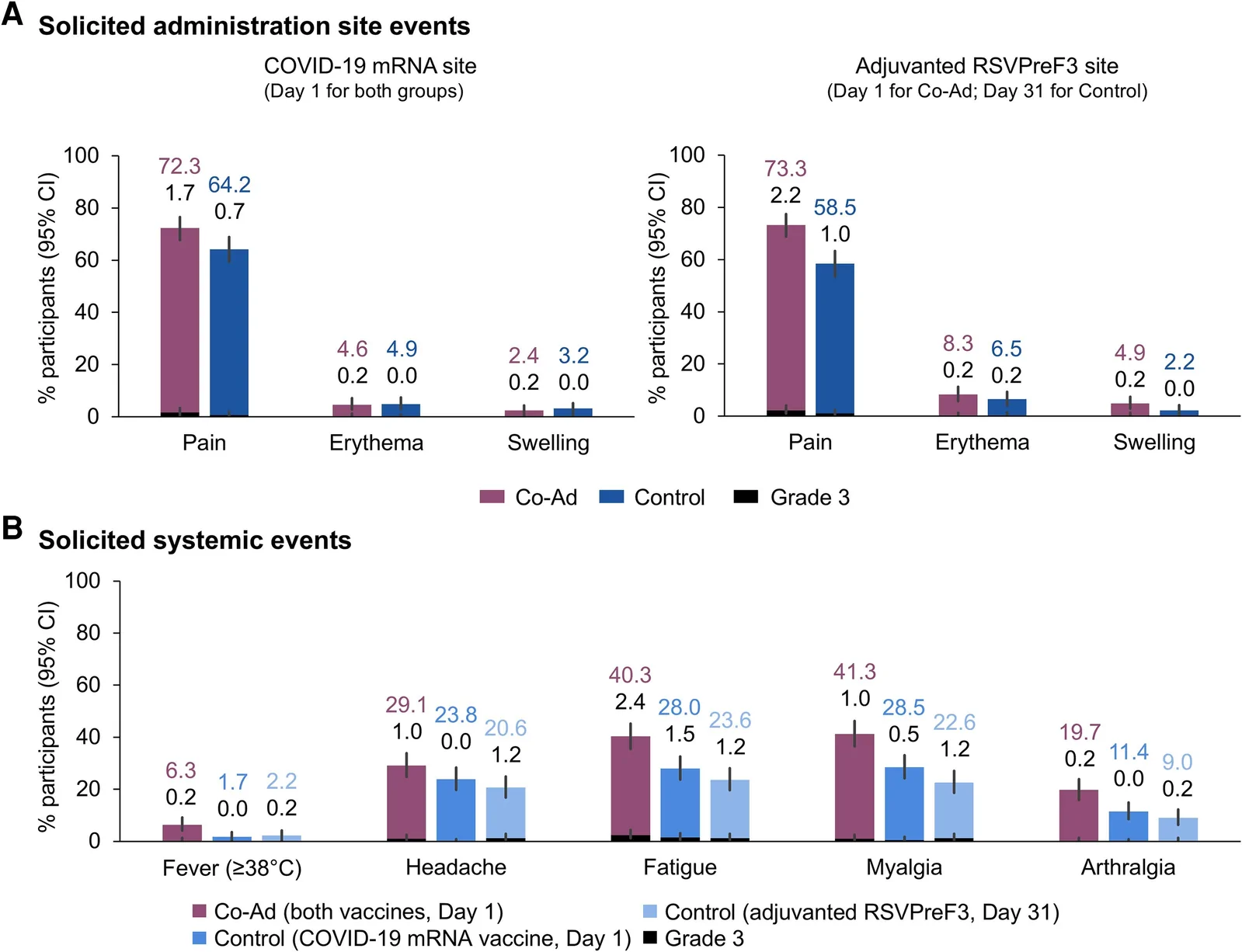
Solicited events with onset within 4 days after adjuvanted RSVPreF3 and coronavirus disease 2019 (COVID-19) mRNA vaccine coadministration or sequential administration, exposed population (with available results). Co-Ad, group of participants receiving a single dose of a COVID-19 mRNA vaccine and a single dose of AS01_E_-adjuvanted respiratory syncytial virus (RSV) prefusion F protein-based vaccine (adjuvanted RSVPreF3) at Day 1; Control, group of participants receiving a single dose of a COVID-19 mRNA vaccine at Day 1, followed by a single dose of adjuvanted RSVPreF3 at Day 31. Notes: Fever was defined by a temperature ≥38.0°C. Grade 3 solicited events were defined by solicited events that prevent normal everyday activities, fever with a temperature >39.0°C, or erythema/swelling with a surface diameter >100 mm. The number of participants with available data from diary cards ranged between 402 and 412 for the different solicited events.

Grade 3 solicited events were reported infrequently across groups, reported by ≤2.2% of participants for any type of solicited administration-site event and ≤2.4% for any type of solicited systemic event at each visit (Figure 6).

The duration of solicited events of any grade was short and comparable between groups, with a median duration of ≤3 days across groups for administration-site events and ≤2 days for systemic events. No participant withdrew from the study due to a solicited event.

In both groups, the frequency of solicited events was generally higher in participants aged 50–59 and 60–69 years when compared with those aged ≥70 years (Supplementary Tables 4 and 5). As the safety analysis was descriptive, these observations should be interpreted with caution.

Overall, 20.9% Co-Ad and 26.0% Control participants (17.1% post-COVID mRNA vaccine; 14.0% postadjuvanted RSVPreF3) reported ≥1 unsolicited AE within 30 days after administration (Table 2); the most common unsolicited AEs by primary System Organ Class allocation were infections and infestations (4.1% and 7.9%), gastrointestinal disorders (4.3% and 6.0%), and general disorders and administration-site conditions (5.5% and 3.8%). No cluster of unsolicited AEs by Preferred Term categorization was observed in either group. Unsolicited AEs considered vaccination-related by the investigator were reported by 9.6% Co-Ad and 7.5% Control participants, the majority being general disorders and administration-site conditions (4.3% and 3.1%) and gastrointestinal disorders (2.6% and 1.7%). Grade 3 unsolicited AEs were reported by 3.6% Co-Ad and 2.6% Control participants; 1.4% and 1.0% of participants reported grade 3 AEs considered vaccination-related.

**Table 2.** Unsolicited Adverse Events, Serious Adverse Events, and Adverse Events of Special Interest After Adjuvanted RSVPreF3 and COVID-19 mRNA Vaccine Coadministration or Sequential Administration, Exposed Population

	Co-Ad N = 417	Control N = 416
Event	n (%)	n (%)
Unsolicited AEs within 30 d following vaccination		
Any grade unsolicited AE	87 (20.9)	108 (26.0)
Any grade unsolicited AE considered related to vaccine^a^	40 (9.6)	31 (7.5)
Grade 3 unsolicited AE	15 (3.6)	11 (2.6)
SAEs up to study end (6 m)		
Any SAE	16 (3.8)	14 (3.4)
SAE considered related to vaccine^a^	3 (0.7)	0 (0)
Fatal SAE	0 (0)	0 (0)
AEs of special interest per protocol up to study end (6 m)		
Any pIMD	0 (0)	1 (0.2)
pIMD considered related to vaccine^a^	0 (0)	0 (0)
Any atrial fibrillation^b^	1 (0.2)	1 (0.2)
Atrial fibrillation considered related to vaccine^a,b^	0 (0)	0 (0)
Any AE leading to study discontinuation	0 (0)	2 (0.5)

For the control group, the data for all visits combined are shown.

Abbreviations: Co-Ad, group of participants receiving a single dose of a COVID-19 mRNA vaccine and a single dose of the AS01_E_-adjuvanted respiratory syncytial virus (RSV) prefusion F protein-based vaccine (adjuvanted RSVPreF3) at Day 1; Control, group of participants receiving a single dose of a coronavirus disease 2019 (COVID-19) mRNA vaccine at Day 1, followed by a single dose of adjuvanted RSVPreF3 at Day 31; N, number of participants in the exposed population; n (%), number (percentage) of participants presenting ≥1 type of adverse event (AE) in a given category; SAE, serious AE; pIMD, potential immune-mediated disease.

^a^By investigator assessment.

^b^Nonserious atrial fibrillation events were recorded within 30 d postvaccination.

Up to study end, 16 (3.8%) Co-Ad and 14 (3.4%) Control participants reported ≥1 SAE (Table 2). Three SAEs in the Co-Ad group (0.7%) were considered vaccination-related by the investigator: 1 Herpes zoster case (reported 7 days after coadministration, recovered/resolved), and 2 cardiac failure cases (1 case reported 2 days after coadministration, recovered/resolved; 1 case reported 24 days after coadministration, not recovered/not resolved at study end, considered as a chronic disease by the investigator). Following sponsor medical review, these events were considered as not related to either study intervention due to the lack of biological plausibility and possible alternate etiologies. No fatalities occurred during the study.

One pIMD (serious pericarditis) in the Control group and 1 serious AF event in each group were reported up to study end. Of note, 2 events of atrial flutter (1 serious and 1 nonserious event) were reported in the same participant with pericarditis in the Control group. Per protocol, atrial flutter was not considered an AESI; however, these events were considered as AESIs by the investigator. None of these events were considered vaccination-related by the investigator. No neurological demyelinating disorders were reported during the study.

## DISCUSSION

This study demonstrated that coadministration of adjuvanted RSVPreF3 with a COVID-19 mRNA vaccine induced noninferior RSV-A and RSV-B neutralizing antibody responses compared with those elicited by adjuvanted RSVPreF3 after sequential administration of the vaccines. Robust immune responses to both RSV subtypes were observed 1 month after vaccination in both groups. The slightly higher fold rises in the Control group versus the Co-Ad group may reflect their generally lower baseline neutralizing titers rather than meaningful differences in vaccine-induced responses between both groups.

For the COVID-19 mRNA vaccine, the primary noninferiority criterion for SARS-CoV-2 neutralizing titers was marginally missed in the per-protocol set; however, this is not expected to have clinically meaningful impact on COVID-19 vaccine-induced protection. Supporting this, a sensitivity analysis in the larger exposed population showed an upper limit of the 95% CI of the adjusted GMT ratio <1.50, suggesting that coadministration performed comparably with sequential administration. The exposed population is arguably more reflective of real-world conditions. Moreover, in spite of the likely prior exposure of participants to the Omicron XBB.1.5 variant, SARS-CoV-2 neutralizing titers rose more than 4-fold in both groups.

Solicited events were more frequent with vaccine coadministration than with sequential administration. Although the observed frequencies remained within the ranges of the reactogenicity profile described in the product information leaflet, some participants may consider the higher frequency of solicited events to be relevant. Nonetheless, solicited events were mostly mild or moderate, short-lived, and did not lead to treatment or study discontinuation. Unsolicited AEs and SAEs were generally balanced between the 2 groups and in line with expectations for adjuvanted RSVPreF3 [14–16], and showed no clustering of events. Overall, adjuvanted RSVPreF3 showed a comparable and acceptable safety profile whether administered sequentially or together with a COVID-19 mRNA vaccine.

Notably, voluntary withdrawals and losses to follow-up were approximately twice as high in the Control group compared with the Co-Ad group, likely because more visits were required; in real-world settings, this difference could be even greater. This observation highlights the potential benefit of coadministration, which may improve vaccination uptake by reducing the number of required visits.

The study's strengths lie in its relatively large sample size and its randomized and multicountry design. Moreover, because all participants had received at least 1 COVID-19 mRNA vaccine before study initiation and we did not exclude individuals with prior SARS-CoV-2 exposure, the study population likely encompassed diverse SARS-CoV-2 exposure histories (prior vaccination and infection with different variants and at varying times). This heterogeneity enhances the real-world relevance of our findings.

Several limitations warrant consideration. Enrollment was restricted to high-income countries, and while individuals with certain chronic conditions were enrolled to reflect real-world demographics, immunocompromised adults were excluded. In addition, most participants self-identified as White, which may limit the generalizability of our findings. Immunogenicity assessments were confined to neutralizing antibodies, and although these are a key component of protective immunity against RSV and SARS-CoV-2, cellular immune responses and other potentially relevant immune parameters were not assessed. The open-label design may have introduced reporting bias in safety outcomes; however, as immunogenicity assessments were conducted in a blinded manner and safety endpoints were descriptive, this limitation is unlikely to impact the overall interpretation of the results. Next, the study was not powered for age-stratified comparisons, limiting the precision of age-specific immunogenicity and reactogenicity estimates. Lastly, the study used a COVID-19 mRNA vaccine that has since been updated. Nonetheless, as interchangeability among mRNA-based vaccines that share similar designs and mechanisms of action is supported in the literature [31–36] and by public health agencies [37–40], our findings provide a plausible basis for extrapolation to other COVID-19 vaccines.

In conclusion, the study findings support the coadministration of adjuvanted RSVPreF3 with a COVID-19 mRNA vaccine, demonstrating robust immune responses and an acceptable safety profile comparable with sequential administration. Overall, coadministration of the vaccines may streamline vaccination efforts and support higher vaccine uptake.

## Supplementary Material

ciag344_Supplementary_Data

## Supplementary Data

Supplementary materials are available at *Clinical Infectious Diseases* online. Consisting of data provided by the authors to benefit the reader, the posted materials are not copyedited and are the sole responsibility of the authors, so questions or comments should be addressed to the corresponding author.
