# Supplementary material for: Immunogenicity and Safety of Coadministration of AS01E-Adjuvanted Respiratory Syncytial Virus Prefusion F Protein Vaccine and a COVID-19 mRNA Vaccine in Adult s Aged ≥50 Years: A Phase 3, Randomized, Noninferiority Trial
Source: Clin Infect Dis. 2026 Jun 4;83(4):e867–77. doi: 10.1093/cid/ciag344 (PMC13715961; doi:10.1093/cid/ciag344)
Supplement: Supplementary file 1 [file ciag344_supplementary_data.docx]

# Immunogenicity and safety of co-administration of AS01_E_-adjuvanted respiratory syncytial virus prefusion F protein vaccine and a COVID-19 mRNA vaccine in adults aged ≥50 years: a phase 3, randomized, non-inferiority trial

# Authors: Marc Bonten^1,2^, Brandon Essink^3^, Nikita Hanning^4^, Isabel Leroux-Roels^5^, Cristina Masuet-Aumatell^6^, Silvina Natalini Martinez^7^, Hiwot Amare Hailemariam^8^, Ghassem Jandari^8^, Paulia Kwo Ton Ge Jua^8^, Charlotte Servais^9^, Sofía Valenciano^8*^, Archana M Jastorff^8^, Vicky Cárdenas^10^, Marie Van der Wielen^8^

# Affiliations:

^1^Julius Center for Health Sciences and Primary Care, University Medical Centre Utrecht, Utrecht University, Utrecht, the Netherlands

^2^European Clinical Research Alliance on Infectious Diseases (ECRAID), Utrecht, the Netherlands

^3^Velocity Clinical Research, Omaha, NE, United States

^4^Centre for the Evaluation of Vaccination, Vaccine and Infectious Disease Institute (VAXINFECTIO), University of Antwerp, Antwerp, Belgium

^5^Center for Vaccinology, Ghent University and Ghent University Hospital, Ghent, Belgium

^6^Preventive Medicine Department, University Hospital Bellvitge, IDIBELL, L’Hospitalet de Llobregat Barcelona, Spain

^7^Hospital Universitario HM Puerta del Sur, Unidad de Investigación de Vacunas, Instituto de Investigación Sanitaria HM, Madrid, Spain

^8^GSK, Wavre, Belgium

^9^GSK, Rixensart, Belgium

^10^GSK, Rockville, MD, United States

# *Corresponding author:

Sofía Valenciano

GSK, Wavre, Belgium

Email: [sofia.8.valenciano@gsk.com](mailto:sofia.8.valenciano@gsk.com)

**Alternate corresponding author:**

Vicky Cárdenas

GSK, Rockville, MD, United States

E-mail: [vicky.x.cardenas@gsk.com](mailto:vicky.x.cardenas@gsk.com)

# Supplementary Material

# Supplementary Methods

## Eligibility criteria

Inclusion criteria

Participants were eligible to be included in the study only if all of the following criteria applied:

- Participants, who, in the opinion of the investigator, could and would comply with the requirements of the protocol (e.g., completion of the diary cards, return for follow-up visits).

*Note: In case of physical incapacity that would preclude the self-completion of the diary cards, either site staff could assist the participant (for activities performed during site visits) or the participant could assign a caregiver to assist him/her with this activity (for activities performed at home). However, at no time, the site staff or caregiver could evaluate the participant’s health status while answering diaries or make decisions on behalf of the participant.*

- Written or witnessed informed consent obtained from the participant (participant had to be able to understand the informed consent) prior to performance of any study-specific procedure.
- A male/female aged ≥50 years at the time of the first study intervention administration.
- Participants who were medically stable in the opinion of the investigator at the time of first vaccination. Participants with chronic stable medical conditions with or without specific treatment, such as diabetes, hypertension or cardiac disease, were allowed to participate in this study if considered by the investigator as medically stable.

*A stable medical condition was defined as disease not requiring significant change in therapy or hospitalization for worsening disease during the three months before enrollment.*

- Participants had to live in the general community or in an assisted-living facility that provided minimal assistance, such that the participant was primarily responsible for self-care and activities of daily living.
- Participants who had received previously a severe acute respiratory syndrome coronavirus 2 (SARS-CoV-2) vaccine, that was administered at least three months prior to study vaccination.
- Female participants of non-childbearing potential could be enrolled in the study. Non‑childbearing potential is defined as hysterectomy, bilateral oophorectomy, bilateral salpingectomy, and post-menopause.
- Female participants of childbearing potential could be enrolled in the study if the participant.
- had practiced adequate contraception from one month prior to study intervention administration and agreed to continue adequate contraception for at least one month after the last vaccination.
- had a negative pregnancy test on the day of and prior to study intervention administration.

Exclusion criteria

Participants were excluded from the study if any of the following criteria applied:

*Medical conditions*

- History of any reaction or hypersensitivity likely to be exacerbated by any component of the study interventions, including a known history of severe allergic reaction (e.g., anaphylaxis).
- Any confirmed or suspected immunosuppressive or immunodeficient condition, resulting from disease (e.g., current malignancy, human immunodeficiency virus) or immunosuppressive/cytotoxic therapy (e.g., medication used during cancer chemotherapy, organ transplantation, or to treat autoimmune disorders), based on medical history and physical examination (no laboratory testing required).
- Any history of myocarditis or pericarditis.
- Recurrent history or uncontrolled neurological disorders or seizures. Participants with medically-controlled active or chronic neurological diseases could be enrolled in the study as per investigator assessment, provided that their condition would allow them to comply with the requirements of the protocol (e.g., completion of diary cards, attend regular phone calls/study site visits).
- Serious or unstable chronic illness.
- Any history of dementia or any medical condition that moderately or severely impaired cognition.

*Note: If deemed necessary for clinical evaluation, the investigator could use tools such as Mini-Mental State Examination (MMSE), Mini-Cog or Montreal Cognitive Assessment (MoCA) to determine cognition levels of the participant.*

- Significant underlying illness that in the opinion of the investigator would be expected to prevent completion of the study (e.g., life-threatening disease likely to limit survival up to study end).
- Any medical condition that in the judgment of the investigator would make intramuscular injection unsafe.
- Any serious adverse event attributed to a previous dose of the SARS-CoV-2 mRNA vaccine.
- Any other clinical condition that, in the opinion of the investigator, might have posed additional risk to the participant due to participation in the study.
- Recent SARS-CoV-2 infection within three months prior to the coronavirus disease 2019 (COVID-19) vaccine dose administration. Timelines had to be determined from symptoms onset or positive COVID-19 test (if infection was asymptomatic).

*Prior/concomitant therapy*

- Use of any investigational or non-registered product (drug, vaccine or invasive medical device) other than the study interventions during the period beginning 30 days before the first dose of study interventions and ending 30 days after the last vaccine administration, or their planned use during the study period.
- Planned administration of a vaccine in the period starting 30 days before the first dose and ending 30 days after the last dose of study intervention(s) administration*, with the exception of inactivated and subunit influenza vaccines which could be administered up to 14 days before or from 14 days after the study vaccination.

**If emergency mass vaccination for an unforeseen public health threat (e.g., a pandemic) was recommended and/or organized by public health authorities outside the routine immunization program, the time period described above could be reduced if necessary for that vaccine, provided it was used according to the local governmental recommendations and Sponsor was notified.*

- Chronic administration of immune-modifying drugs (defined as more than 14 consecutive days in total) and/or planned use of long-acting immune-modifying treatments at any time up to the last blood sampling visit.
- Up to three months prior to the study intervention administration:
- For corticosteroids, this meant *prednisone* equivalent ≥20 mg/day. Inhaled and topical steroids were allowed.
- Administration of immunoglobulins and/or any blood products or plasma derivatives.
- Up to six months prior to study intervention administration: long-acting immune‑modifying drugs including among others immunotherapy (e.g., tumor necrosis factor inhibitors), monoclonal antibodies and antitumoral medication.
- Administration of any SARS-CoV-2 vaccine during the three months preceding the study COVID-19 mRNA vaccine administration.
- Previous vaccination with licensed or investigational RSV vaccine.

*Prior/concurrent clinical study experience*

- Concurrently participating in another clinical study, at any time during the study period, in which the participant had been or would have been exposed to an investigational or a non-investigational intervention (drug/invasive medical device).

*Other exclusion criteria*

- Pregnant or lactating female participant.
- Female participant planning to become pregnant or planning to discontinue contraceptive precautions.
- History of chronic alcohol consumption and/or drug abuse as deemed by the investigator that rendered the potential participant unable/unlikely to provide accurate safety reports or comply with study procedures.
- Participation of any study personnel or their immediate dependents, family, or household members.
- Planned move during the study conduct that prohibited participation until study end.
- Bedridden participants.

## Enrollment rules

Participants were enrolled in three age categories to reflect the age distribution in the general population, with a balance between males and females. Approximately 30% of participants needed to be aged 50–59 years, 30% of participants 60–69 years, and 25% of participants ≥70 years; the remaining 15% could be distributed freely across the three age categories. Approximately 40% of participants needed to be enrolled from each sex; the remaining 20% could be distributed freely between the two sexes.

## Randomization procedures

A randomization schedule was used to allocate participants in a balanced, centralized, and stratified manner in the two groups. The schedule was generated using the GSK validated RANDALL NG randomization software. It employed a permuted block design, with a 1:1 treatment allocation within each block, and including age with three strata (50–59, 60–69 or ≥70 years) as a stratification factor, to remain balanced across age groups as enrollment proceeded. The randomization schedule was shared across study centers via a central randomization system, using an automated Internet-based system (randomization and trial supply management [RTSM]). When a participant was enrolled and an identification number was assigned, RTSM determined the group and provided the study intervention number to be used for the first dose. For subsequent dosing, RTSM provided the study intervention number(s) as needed.

## Sample size determination

Assuming a 13% non‑evaluable rate among enrolled participants up to two months post-vaccination, a target of 830 enrolled participants (415 per group) was set to reach 361 evaluable participants per group for the primary objective. With 361 evaluable participants per group, and to control the 1-sided type I error at 2.5% for each primary objective (i.e., alpha of 0.025 per endpoint), the power to demonstrate immunological non-inferiority with the specified non-inferiority margins in the Co-Ad group compared to the Control group in terms of RSV-A, RSV-B, and SARS-CoV-2 neutralizing titers would be 99.6% for both RSV subtypes, and 90.9% for SARS-CoV-2.

## Pseudotyped virus neutralization assay

The SARS-CoV-2 pseudotyped virus neutralization assay evaluated the level of SARS-CoV-2 neutralization titers present in the human serum samples. Pseudotyped virus particles were made from a modified Vesicular Stomatitis Virus (VSVΔG) backbone and beared the spike glycoprotein of the SARS-CoV-2 variant of interest (Omicron XBB.1.5). In addition, the pseudoparticles contained a luciferase reporter gene to allow detection and quantification of VSVΔG internalized by the target cells. The intensity of luminescence was quantified in relative luminescence units, which is inversely proportional to the neutralization titer. The neutralizing titer of a serum sample was calculated as the reciprocal serum dilution corresponding to the 50% signal reduction (NT50) for that sample.

# Supplementary Results

## Supplementary Table 1. Baseline characteristics of the participants, RSV and COVID-19 mRNA per-protocol sets for primary analysis

| Characteristic | RSV PPS | | COVID-19 mRNA PPS | |
| --- | --- | --- | --- | --- |
|  | Co-Ad | Control | Co-Ad | Control |
|  | N=382 | N=364 | N=380 | N=381 |
| Mean (SD) age at vaccination, y | 63.6 (8.3) | 63.8 (8.5) | 63.5 (8.2) | 63.7 (8.4) |
| Age category, n (%) |  |  |  |  |
| 50–59 y | 142 (37.2) | 135 (37.1) | 141 (37.1) | 143 (37.5) |
| 60–69 y | 138 (36.1) | 127 (34.9) | 138 (36.3) | 134 (35.2) |
| ≥70 y | 102 (26.7) | 102 (28.0) | 101 (26.6) | 104 (27.3) |
| ≥80 y | 13 (3.4) | 8 (2.2) | 12 (3.2) | 8 (2.1) |
| Female sex, n (%) | 202 (52.9) | 194 (53.3) | 202 (53.2) | 205 (53.8) |
| Race, n (%) |  |  |  |  |
| Black or African American | 29 (7.6) | 22 (6.0) | 28 (7.4) | 28 (7.3) |
| White | 345 (90.3) | 335 (92.0) | 344 (90.5) | 346 (90.8) |
| Other^a^ | 8 (2.1) | 7 (1.9) | 8 (2.1) | 7 (1.8) |
| Ethnicity, n (%) |  |  |  |  |
| Hispanic or Latino | 28 (7.3) | 15 (4.1) | 28 (7.4) | 20 (5.2) |
| Not Hispanic or Latino | 351 (91.9) | 346 (95.1) | 349 (91.8) | 358 (94.0) |
| Not reported | 3 (0.8) | 3 (0.8) | 3 (0.8) | 3 (0.8) |
| Country, n (%) |  |  |  |  |
| Belgium | 114 (29.8) | 114 (31.3) | 114 (30.0) | 117 (30.7) |
| The Netherlands | 61 (16.0) | 69 (19.0) | 61 (16.1) | 67 (17.6) |
| Spain | 90 (23.6) | 77 (21.2) | 89 (23.4) | 80 (21.0) |
| United States | 117 (30.6) | 104 (28.6) | 116 (30.5) | 117 (30.7) |

PPS, per-protocol set; Co-Ad, group of participants receiving a single dose of a COVID-19 mRNA vaccine and a single dose of AS01_E_-adjuvanted respiratory syncytial virus (RSV) prefusion F protein-based vaccine (adjuvanted RSVPreF3) at Day 1; Control, group of participants receiving a single dose of a COVID-19 mRNA vaccine at Day 1, followed by a single dose of adjuvanted RSVPreF3 at Day 31; N, number of participants in the PPS; SD, standard deviation; y, years; n (%), number (percentage) of participants in a given category.

^a^Includes American Indian or Alaska Native, Asian, multiple race categories, or race not reported.

## Supplementary Table 2. Underlying medical conditions based on MedDRA classification, exposed population

| **Pre-existing condition, n (%)**  **SOC**  HLT  PT | **Co-Ad**  **N=417** | **Control**  **N=416** |
| --- | --- | --- |
| Any | 383 (91.8) | 395 (95.0) |
| **Metabolism and nutrition disorders** | **206 (49.4)** | **199 (47.8)** |
| Elevated cholesterol | 104 (24.9) | 93 (22.4) |
| Hypercholesterolaemia | 104 (24.9) | 93 (22.4) |
| Diabetes mellitus (incl subtypes) | 69 (16.5) | 57 (13.7) |
| Type 2 diabetes mellitus | 67 (16.1) | 57 (13.7) |
| **Vascular disorders** | **192 (46.0)** | **176 (42.3)** |
| Vascular hypertensive disorders NEC | 181 (43.4) | 162 (38.9) |
| Hypertension | 178 (42.7) | 161 (38.7) |
| **Musculoskeletal and connective tissue disorders** | **136 (32.6)** | **109 (26.2)** |
| Osteoarthropathies | 65 (15.6) | 48 (11.5) |
| Osteoarthritis | 60 (14.4) | 44 (10.6) |
| **Infections and infestations** | **112 (26.9)** | **111 (26.7)** |
| Coronavirus infections | 79 (18.9) | 63 (15.1) |
| COVID-19 | 72 (17.3) | 57 (13.7) |
| **Surgical and medical procedures** | **104 (24.9)** | **103 (24.8)** |
| **Psychiatric disorders** | **97 (23.3)** | **102 (24.5)** |
| Depressive disorders | 44 (10.6) | 45 (10.8) |
| **Gastrointestinal disorders** | **90 (21.6)** | **104 (25.0)** |
| Gastrointestinal atonic and hypomotility disorders NEC | 52 (12.5) | 54 (13.0) |
| Gastrooesophageal reflux disease | 50 (12.0) | 52 (12.5) |
| **Respiratory, thoracic and mediastinal disorders** | **86 (20.6)** | **105 (25.2)** |
| Bronchospasm and obstruction | 56 (13.4) | 69 (16.6) |
| **Immune system disorders** | **91 (21.8)** | **74 (17.8)** |
| Atopic disorders | 52 (12.5) | 53 (12.7) |
| Seasonal allergy | 52 (12.5) | 53 (12.7) |
| **Nervous system disorders** | **72 (17.3)** | **75 (18.0)** |
| **Cardiac disorders** | **66 (15.8)** | **52 (12.5)** |
| **Reproductive system and breast disorders** | **45 (10.8)** | **59 (14.2)** |

Pre-existing medical conditions were obtained by interviewing the participant and/or reviewing the participant’s medical records. Classification was done according to Medical Dictionary for Regulatory Activities (MedDRA) Terminology: System Organ Class (SOC), high-level term (HLT), and preferred term (PT). This summary table presents baseline medical conditions categorized by SOC, HLT, and PT with a frequency of ≥10.0% in at least one of the two study groups.

n (%), number (percentage) of participants in the indicated category; N, number of participants in the exposed population; Co-Ad, group of participants receiving a single dose of a COVID-19 mRNA vaccine and a single dose of AS01_E_-adjuvanted respiratory syncytial virus (RSV) prefusion F protein-based vaccine (adjuvanted RSVPreF3) at Day 1; Control, group of participants receiving a single dose of a COVID-19 mRNA vaccine at Day 1, followed by a single dose of adjuvanted RSVPreF3 at Day 31; NEC, not elsewhere classified.

## Supplementary Table 3. Solicited systemic events with onset within four days after adjuvanted RSVPreF3 and COVID-19 mRNA vaccine co-administration or sequential administration, per participant, exposed population (with available results)

| **Event, n (%)** | **Co-Ad**  **N=412** | **Control**  **N=411** |
| --- | --- | --- |
| Fever |  |  |
| Any | 26 (6.3) | 16 (3.9) |
| Grade 3 | 1 (0.2) | 1 (0.2) |
| Headache |  |  |
| Any | 120 (29.1) | 135 (32.8) |
| Grade 3 | 4 (1.0) | 5 (1.2) |
| Fatigue |  |  |
| Any | 166 (40.3) | 153 (37.2) |
| Grade 3 | 10 (2.4) | 11 (2.7) |
| Myalgia |  |  |
| Any | 170 (41.3) | 156 (38.0) |
| Grade 3 | 4 (1.0) | 7 (1.7) |
| Arthralgia |  |  |
| Any | 81 (19.7) | 69 (16.8) |
| Grade 3 | 1 (0.2) | 1 (0.2) |

Co-Ad, group of participants receiving a single dose of a COVID-19 mRNA vaccine and a single dose of AS01_E_-adjuvanted respiratory syncytial virus (RSV) prefusion F protein-based vaccine (adjuvanted RSVPreF3) at Day 1; Control, group of participants receiving a single dose of a COVID-19 mRNA vaccine at Day 1, followed by a single dose of adjuvanted RSVPreF3 at Day 31; N, number of participants in the exposed population with available results; n (%), number (percentage) of participants presenting with ≥1 symptom in a given category.

Notes: Participants in the Control group who experienced a given event after each vaccine were counted once. Fever was defined by a temperature ≥38.0°C. Grade 3 solicited events were defined by events that prevent normal everyday activities or fever with a temperature >39.0°C.

## Supplementary Table 4. Solicited administration-site events with onset within four days after adjuvanted RSVPreF3 and COVID-19 mRNA vaccine co-administration or sequential administration, by age category, exposed population (with available results)

| **Event, n (%)** | **Co-Ad** | | | **Control** | | |
| --- | --- | --- | --- | --- | --- | --- |
|  | **50–59 years** | **60–69 years** | **≥70 years** | **50–59 years** | **60–69 years** | **≥70 years** |
| **COVID-19 mRNA dosing** | **N=151** | **N=148** | **N=112** | **N=157** | **N=144** | **N=110** |
| Pain | 126 (83.4) | 107 (72.3) | 64 (57.1) | 109 (69.4) | 98 (68.1) | 57 (51.8) |
| Erythema | 3 (2.0) | 6 (4.1) | 10 (8.9) | 5 (3.2) | 9 (6.3) | 6 (5.5) |
| Swelling | 4 (2.6) | 4 (2.7) | 2 (1.8) | 4 (2.5) | 6 (4.2) | 3 (2.7) |
| **Adjuvanted RSVPreF3 dosing** | **N=151** | **N=148** | **N=113** | **N=152** | **N=142** | **N=108** |
| Pain | 119 (78.8) | 116 (78.4) | 67 (59.3) | 110 (72.4) | 83 (58.5) | 42 (38.9) |
| Erythema | 8 (5.3) | 15 (10.1) | 11 (9.7) | 14 (9.2) | 7 (4.9) | 5 (4.6) |
| Swelling | 8 (5.3) | 7 (4.7) | 5 (4.4) | 5 (3.3) | 4 (2.8) | 0 (0.0) |

Co-Ad, group of participants receiving a single dose of a COVID-19 mRNA vaccine and a single dose of AS01_E_-adjuvanted respiratory syncytial virus (RSV) prefusion F protein-based vaccine (adjuvanted RSVPreF3) at Day 1; Control, group of participants receiving a single dose of a COVID-19 mRNA vaccine at Day 1, followed by a single dose of adjuvanted RSVPreF3 at Day 31; N, number of participants in the exposed population with available results; n (%), number (percentage) of participants presenting with ≥1 symptom in a given category.

## Supplementary Table 5. Solicited systemic events with onset within four days after adjuvanted RSVPreF3 and COVID-19 mRNA vaccine co-administration or sequential administration, by age category, exposed population (with available results)

| **Event, n (%)** | **Co-Ad (both vaccines)** | | | **Control (COVID-19 mRNA)** | | | **Control (Adjuvanted RSVPreF3)** | | |
| --- | --- | --- | --- | --- | --- | --- | --- | --- | --- |
|  | **50–59 years**  **N=151** | **60–69 years**  **N=148** | **≥70 years**  **N=113** | **50–59 years**  **N=157** | **60–69 years**  **N=144** | **≥70 years**  **N=110** | **50–59 years**  **N=152** | **60–69 years**  **N=142** | **≥70 years**  **N=108** |
| Fever | 13 (8.6) | 7 (4.7) | 6 (5.3) | 5 (3.2) | 1 (0.7) | 1 (0.9) | 8 (5.3) | 0 (0.0) | 1 (0.9) |
| Headache | 58 (38.4) | 45 (30.4) | 17 (15.0) | 52 (33.1) | 32 (22.2) | 14 (12.7) | 44 (28.9) | 20 (14.1) | 19 (17.6) |
| Fatigue | 73 (48.3) | 55 (37.2) | 38 (33.6) | 54 (34.4) | 39 (27.1) | 22 (20.0) | 56 (36.8) | 24 (16.9) | 15 (13.9) |
| Myalgia | 74 (49.0) | 63 (42.6) | 33 (29.2) | 58 (36.9) | 41 (28.5) | 18 (16.4) | 47 (30.9) | 31 (21.8) | 13 (12.0) |
| Arthralgia | 35 (23.2) | 32 (21.6) | 14 (12.4) | 24 (15.3) | 15 (10.4) | 8 (7.3) | 17 (11.2) | 13 (9.2) | 6 (5.6) |

Co-Ad, group of participants receiving a single dose of a COVID-19 mRNA vaccine and a single dose of AS01_E_-adjuvanted respiratory syncytial virus (RSV) prefusion F protein-based vaccine (adjuvanted RSVPreF3) at Day 1; Control, group of participants receiving a single dose of a COVID-19 mRNA vaccine at Day 1, followed by a single dose of adjuvanted RSVPreF3 at Day 31; N, number of participants in the exposed population with available results; n (%), number (percentage) of participants presenting with ≥1 symptom in a given category.

## Supplementary Figure 1. RSV-A and RSV-B neutralizing titers after adjuvanted RSVPreF3 and COVID-19 mRNA vaccine co-administration or sequential administration, by age category, RSV and COVID-19 mRNA per-protocol sets

RSV, respiratory syncytial virus; Co-Ad, group of participants receiving a single dose of a COVID-19 mRNA vaccine and a single dose of AS01_E_-adjuvanted RSV prefusion F protein-based vaccine (adjuvanted RSVPreF3) at Day 1; Control, group of participants receiving a single dose of a COVID-19 mRNA vaccine at Day 1, followed by a single dose of adjuvanted RSVPreF3 at Day 31; GMT, geometric mean titer; ED60, Estimated Dilution 60; CI, confidence interval; MGI, mean geometric increase post-vaccination compared to pre-vaccination; NT50, serum dilution corresponding to 50% signal reduction.

Note: Participant numbers in the per-protocol sets with available results were as follows (GMTs pre-/post-vaccination; MGIs both timepoints):

RSV per-protocol set (50–59 years, 60–69 years, ≥70 years): Co‑Ad 151/143 (MGI 142), 145/140 (MGI 138), 110/105 (MGI 102); Control 144/135 (MGI 135), 138/127 (MGI 127), 107/103 (MGI 102).

COVID‑19 mRNA per-protocol set (50–59 years, 60–69 years, ≥70 years): Co‑Ad 151/142 (MGI 141), 145/140 (MGI 138), 109/105 (MGI 101); Control 155/144 (MGI 143), 141/138 (MGI 134), 108/107 (MGI 104).
